# The Central THαβ Immunity Associated Cytokine: IL-10 Has a Strong Anti-Tumor Ability Toward Established Cancer Models *In Vivo* and Toward Cancer Cells *In Vitro*

**DOI:** 10.3389/fonc.2021.655554

**Published:** 2021-04-12

**Authors:** Wan-Chung Hu

**Affiliations:** ^1^ Genomics Research Center, Academia Sinica, Taipei, Taiwan; ^2^ Taipei Tzu Chi Hospital, Buddhist Tzu Chi Medical Foundation, New Taipei City, Taiwan

**Keywords:** Tr1, immunotherapy, IL-10, IL-15, viral immune response

## Abstract

Immunotherapy is a promising new approach for cancer treatment. In this study, I propose to use the THαβ-mediated immune response for cancer treatment. The THαβ-mediated immune response is activated by IL-10 and IL-15. Thus, I used IL-10 and-15 as therapeutic agents in the 4T1 cell line, which is a mouse cell line of breast cancer, and the NXS2 cell line, which is a mouse cell line of neuroblastoma. Cells from 4T1 and NXS2 were subcutaneously inoculated in wild type BALB/c female mice and AJ mice, respectively, and administered cytokines or an antibody treatment at various dosages. My results showed that IL-10 and IL-15 administration led to reduction in tumor volume and increase in survival. However, traditional TH1 cytokine IFN-γ administration led to increase in tumor volume and decline in survival. Antibody treatment in conjunction with IL-10 was not significantly better than IL-10, due to the expression of GD2 on immune cells. Moreover, an anti-GD2 antibody inhibited the immune cells themselves. Additionally, I found that IL-10 was directly toxic to tumor cells *in vitro*. Thus, I conclude that the THαβ immunological pathway is a good treatment strategy for cancer.

## Introduction

Current cancer treatments, including surgery, radiotherapy, and chemotherapy, have been found to yield unsatisfactory results; however, immunotherapy has emerged as a prospective new treatment strategy for cancer. Although current immunotherapies involving monoclonal antibodies are effective, resistance to monoclonal antibody treatments in cancer patients has been reported. Thus, we need more efficacious ways to activate the host immune system against cancer. Cytokines are important players in activating immune cells, and thus, I can use them as immunostimulants during monoclonal antibody treatment regimens against cancer.

The host immune system has four branches (1–4), namely T-helper (TH)2-, TH17-, TH1-, and THαβ-mediated immune responses. The TH2-mediated immune response is activated against helminths, and its effectors include eosinophils, basophils, mast cells, IgE/IgG4-secreting B cells, and IL-4/IL-5-secreting CD4^+^ T cells. The TH17-mediated immune response is activated against extracellular bacteria, and its effectors include neutrophils, IgA/IgG2-secreting B cells, and TGFβ/IL-6/IL-17-secreting CD4^+^ T cells. The traditional TH1-mediated immune response is activated against intracellular bacteria, and its effectors include macrophages, IgG3-secreting B cells, cytotoxic T cells, and IFN-γ-secreting CD4^+^ T cells. In a previous study, I proposed a new host immunological pathway involving THαβ-mediated immune response(formerly called Tr1), which is activated against viruses, and its effectors include natural killer (NK) cells, IgG1-secreting B cells, cytotoxic T cells, and IL-10/IFN-α/β-secreting CD4^+^ T cells (5).

The THαβ cell-mediated immune response is a newly proposed host immunological pathway. This immune response is a hypersensitivity response involving antibody-dependent cellular cytotoxicity (ADCC). In this immune response, NK cell-mediated ADCC is the major mechanism of eliminating virus-infected cells. Thus, I speculate that this mechanism is also used to eliminate tumor cells with tumor antigens present on the cell surface. Furthermore, other immunological pathways are not suitable candidates for cancer immunotherapy. For example, TH2-mediated immune response involves histamine-mediated physiological expulsion of parasites and does not play a role in the immune response against cancers. However, TH17, along with the key mediator molecule, TGFβ, not only promotes angiogenesis but also suppresses the host immune response by activating Treg cells. TH17 can thus enhance tumor growth. Macrophages are crucial to the traditional TH1-mediated immune response and enhance tumor invasiveness and metastasis. Thus, the traditional TH1-mediated immune response is also not a good candidate for cancer immunotherapy. In this study, I investigated the efficacy of the THαβ-mediated immune response in cancer immunotherapy. We investigated the role of cytokine adjuvants in activating NK cell-mediated ADCC using a 4T1 mouse breast cancer cell line and an NXS2 mouse neuroblastoma cell line for establishing our *in vivo* tumor models (6).

## Materials and Methods

### Cell Culture

4T1 cells and NXS2 cells were obtained from American Type Culture Collection. 4T1 cells were maintained in RPMI 1640 medium, and NXS2 cells were maintained in Dulbecco’s modified Eagle’s medium (Gibco Inc., MA). Cells were passaged every three days.

### Establishment of a Mouse Tumor Model and Treatment Strategies

Balb/c female and AJ male and female mice, aged 6-8 weeks, were purchased from the National Experimental Animal Center of Taiwan. 4T1 and NXS2 cells were subcutaneously inoculated into the right flank of Balb/c female mice and AJ female or male mice, respectively, on day 0. Mice infected with 4T1 cells were intraperitoneally injected with cytokines (human IL-10, 20 µg; mouse IL-15, 15 µg; IFN-γ, 5x10^4^ IU; all purchased from BioLegend Inc., CA) on day 1, 3, 5, 7, and 9. Mice infected with NXS2 cells were intraperitoneally injected with 12. 5 µg of IL-10 for 10 days, with or without 150 µg of 14G2A (anti-GD2 antibody, from Dr. Alice L. Yu’s lab) administered intravenously on day 1, 5, and 9. Tumor volume was measured every week, and the survival rate was also recorded.

### Cell Counting

A pilot study was performed to calculate the *in vitro* toxicity of IL-10 in NXS2 and 4T1 cells. NXS2 or 4T1 cells were seeded in 5-cm^3^ dishes and incubated with or without IL-10 (2 µg/cm^3^ and 4 µg/cm^3^) for 3 days. Then, cells were harvested, and automatic cell counting was performed to estimate the cell number with or without IL-10 treatment.

### alamarBlue Assay

Two hundred microliters of 4T1 cells (1000 cells) were seeded in a 96-well plate overnight and then incubated with various dosages of IL-10 ranging from 2-10 µg/cm^3^ for 2 days. Then, alamarBlue (Thermo Fisher Scientific Inc., MA) was added to wells for estimation of viable cells. Viable cell counts for NXS2 cells were estimated by incubating per well 2000 NXS2 cells, 1500 RAW cells as positive control, or 1500 NIH 3T3 fibroblasts as negative control with IL-10 for 3, 2, and 2 days, respectively.

### Flowcytometry

Flowcytometry (BD Bioscience Inc., NJ) was performed to estimate the expression of Globo H antigen, Fc receptor (CD16, CD32, and CD64), and IL-10 receptor (CD210) on 4T1 and NXS2 cells. Antibodies for the aforementioned antigens were purchased from eBioscience Inc, CA. One microgram of each antibody against the above antigen was added to 2X 10^5^ 4T1 or NXS2 cells and incubated for 30 min. Then, flowcytometry was performed to estimate antigen expression relative to the isotype control.

### GD2 Antigen Induction

Balb/c mice were intraperitoneally injected with 10 µg of IL-10 for 1 or 3 days. Injected mice were then sacrificed, and splenocytes were harvested. The expression level of GD2 antigen on the splenocytes was estimated by flowcytometric analysis using anti-GD2 IgG antibody (from Dr. Alice L. Yu’s lab) and compared to control mice, which were not injected with IL-10. Results were analyzed by using the FlowJo software (Tree Star, Asland, OR).

## Results

### IL-10 Led to Tumor Shrinkage and Prolonged Survival in the 4T1 Cell-Injected Mice

**Figure 1:**
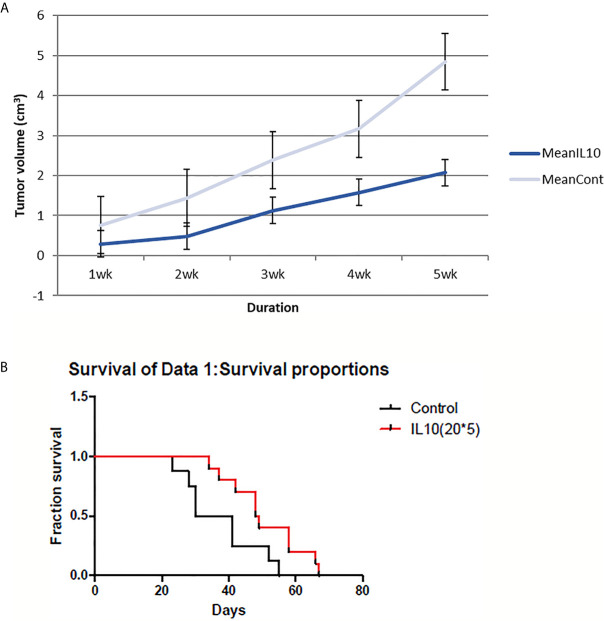
4T1 tumor volume and mice survival graph after IL-10 injection compared to control mice (n=12 each group). **(A)** Tumor volume was measured every week after 4T1 cell inoculation with or without IL-10 treatment. Grey line means control group and blue line means the IL-10 group. IL-10 therapy caused statistically significant tumor volume shrinkage (standard error is used in the graph). **(B)** Mice survival was measured each week after 4T1 cell inoculation with or without IL-10 treatment. Red line means IL-10 treated group and black line means control group. IL-10 treatment significantly prolonged mice survival. (Likelihood P value=0.020, median survival for control group=35.5 days and for IL-10 treated group=48.5 days).

IL-10 had significant effects on both tumor volume shrinkage and survival rate (**Figure 1**). The size of tumors in IL-10-injected mice was much smaller compared with that in control mice. In addition, IL-10 treatment prolonged the survival of treated mice compared with that of control mice. Thus, my findings suggest that IL-10 is a potential candidate for cancer immunotherapy.

### IL-15 Led to Reduction in Tumor Volume and Prolonged Survival of Mice Injected With 4T1 Cells

**Figure 2:**
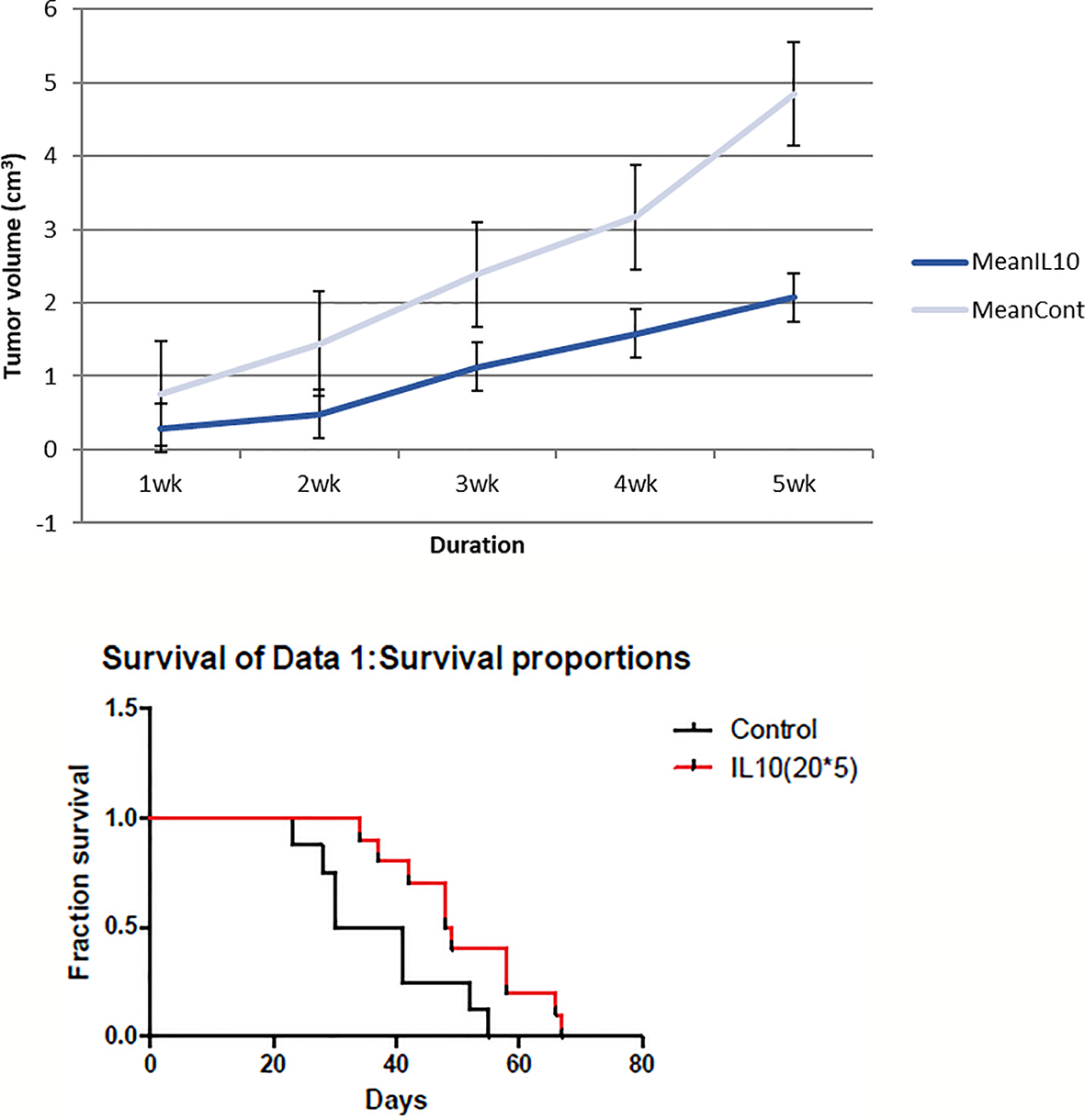
4T1 tumor volume and mice survival graph after IL-15 injection compared to control mice (n=9 each group). **(A)** Tumor volume was measured every week after 4T1 cell inoculation with or without IL-15 treatment. Grey line means control group and blue line means the IL-15 group. IL-15 therapy caused statistically significant tumor volume shrinkage(standard error is used in the graph). **(B)** Mice survival was measured each week after 4T1 cell inoculation with or without IL-15 treatment. Red line means IL-15 treated group and black line means control group. IL-15 treatment significantly prolonged mice survival. (Likelihood P value=0.048, median survival for control group=41 days and for IL-15 treated group=50 days).

The cytokine IL-15 was also found to suppress tumor growth and prolong survival of mice (**Figure 2**). Tumor volumes were significantly lower in IL-15-injected mice compared to that in the control mice, especially in the later stages. IL-15 administration also prolonged survival compared with that of the control mice; mice injected with IL-15 survived 2 weeks more than control mice.

### IFN-γ Led to Increase in Tumor Volume and Reduction in the Survival of Mice Injected With 4T1 Cells

**Figure 3:**
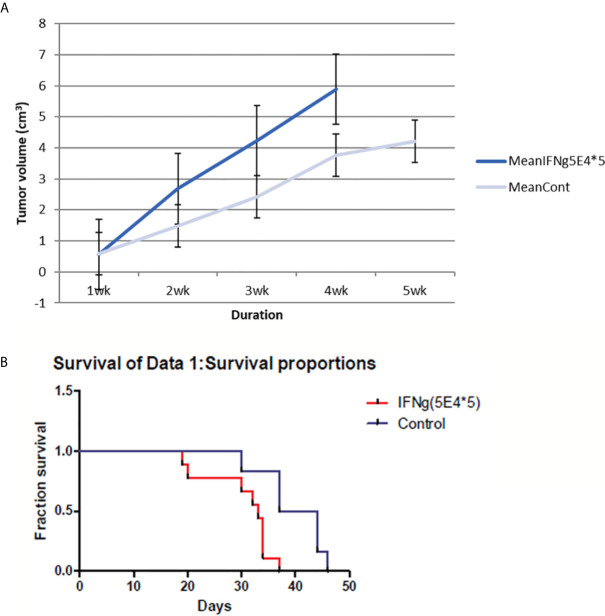
4T1 tumor volume and mice survival graph after IFN-γ injection compared to control mice (n=9 each group). **(A)** Tumor volume was measured every week after 4T1 cell inoculation with or without IFN-γ treatment. Grey line means control group and blue line means the IFN-γ group. IFN-γ therapy caused statistically significant tumor volume enlargement (standard error is used in the graph). **(B)** Mice survival was measured each week after 4T1 cell inoculation with or without IFN-γ treatment. Red line means IFN-γ treated group and blue line means control group. IFN-γ treatment significantly shortened mice survival. (Likelihood P value=0.01, median survival for control group=40.5 days and for IFN-γ treated group=33 days).

Surprisingly, I found an increase in tumor size in IFN-γ-injected mice compared to control mice. In addition, IFN-γ injection reduced the survival rate of treated mice compared to that of control mice (**Figure 3**). Thus, my results suggest that IFN-γ had adverse effects on host immune response against tumors.

### IL-10 and 14G2A Suppressed Tumor Volume and Prolonged the Survival of Mice Injected With NXS2 Cells

**Figure 4:**
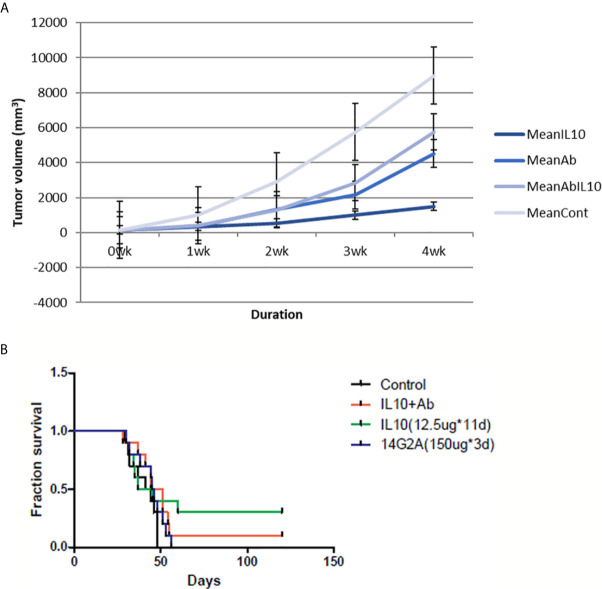
NXS2 tumor volume and mice survival graph after IL-10 injection compared to control mice (n=10). **(A)** Tumor volume was measured every week after NSX2 cell inoculation with or without IL-10 treatment. Grey line means control group, dark blue line means the IL-10 group, light blue line is the 14G2A antibody group, and blue line is the 14G2A antibody plus IL-10 group. IL-10 therapy caused statistically significant tumor volume shrinkage. Antibody alone or antibody plus IL-10 caused mild-moderate tumor volume shrinkage. (standard error is used in the graph). **(B)** Mice survival was measured each week after NXS2 cell inoculation with or without IL-10 treatment. Green line means IL-10 treated group, black line means control group, blue line means 14G2A antibody group, and red line means 14G2A antibody plus IL-10 group. IL-10 treatment most significantly prolonged mice survival.

Administration of IL-10 was also found to suppress tumor volume and prolong the survival of AJ mice injected with NXS2 cells. In addition, 14G2A anti-GD2 antibody also led to reduction in tumor volume and prolonged survival of mice injected with NXS2 cells. However, a combination of IL-10 and 14G2A had a similar effect on tumor volume and mice survival as did 14G2A administration alone. I found that IL-10 alone was more effective than either 14G2A or a combination of 14G2A and IL-10. It is worth noting that IL-10 alone resulted in complete tumor regression in 3 out of 10 AJ mice, and these three mice remained healthy even after the complete 4-month follow-up period. This suggests that IL-10 treatment has a very potent anti-tumor effect compared to currently used cancer therapeutic agents (**Figure 4**).

### IL-10 Reduces Numbers of 4T1 Cells and NXS2 Cells *In Vitro*

**Figure 5:**
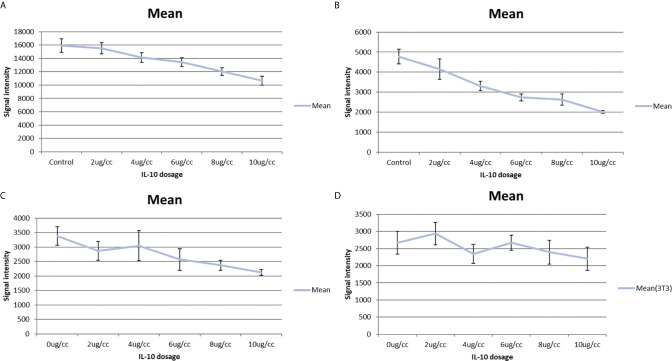
IL-10 *in vitro* toxicity on cell lines shown by alamar blue cell titer assay. **(A)** Alamar blue cell titer results of 4T1 cells treated with varies dosages of IL-10. A dose-response graph is noted and IL-10 has direct *in vitro* toxicity on 4T1 cells. (standard error is used in the graph). **(B)** Alamar blue cell titer results of NXS2 cells treated with varies dosages of IL-10. A dose-response graph is noted and IL-10 has direct *in vitro* toxicity on NXS2 cells. (standard error is used in the graph). **(C)** Alamar blue cell titer results of RAW macrophage cells treated with varies dosages of IL-10. A dose-response graph is noted and IL-10 has direct *in vitro* toxicity on RAW macrophage cells.(Positive control) (standard error is used in the graph). **(D)** Alamar blue cell titer results of NIH 3T3 fibroblast cells treated with varies dosages of IL-10. No dose-response graph is noted and IL-10 has no *in vitro* toxicity on NIH 3T3 fibroblast cells. (Negative control) (standard error is used in the graph).

In a pilot study, I tested the *in vitro* toxicity of IL-10 on NXS2 and 4T1 tumor cells. IL-10 incubation led to reduction in 4T1 and NXS2 cell counts compared with the control treatment (**Tables 1** and **2**). alamarBlue was used to estimate the dose response relationship of IL-10 on the viability of 4T1 and NXS2 cells (**Figure 7**). I found that the decrease in 4T1 and NXS2 cell numbers depended on the concentration of IL-10. A higher IL-10 concentration resulted in a higher degree of tumor cell suppression. This effect was also seen in RAW cells, the mouse macrophage cell line; however, IL-10 had no effect on NIH 3T3 fibroblast cell line (**Figure 5**). Thus, my results suggest that IL-10 was selectively toxic to tumor cell lines.

**Table 1:** NXS2 cell counts after IL-10 treatment for three days(three replicates: A,B,C, P value=0.02, sample means are different).

NXS2	A	B	C	Mean
Control	680000	680000	640000	670000
IL10 2ug/cc	580000	660000	580000	610000
IL10 4ug/cc	490000	580000	560000	540000

**Table 2:** 4T1 cell counts after IL-10 treatment for three days(three replicates:A,B,C, P value=0.01,sample means are different).

4T1	A	B	C	Mean
Control	770000	510000	690000	656666.7
IL10 4ug/cc	490000	450000	360000	433333.3

### Fc Receptor (CD16/32/64) and IL-10 Receptor (CD210) Expression on NXS2 and 4T1 Cells

**Figure 6:**
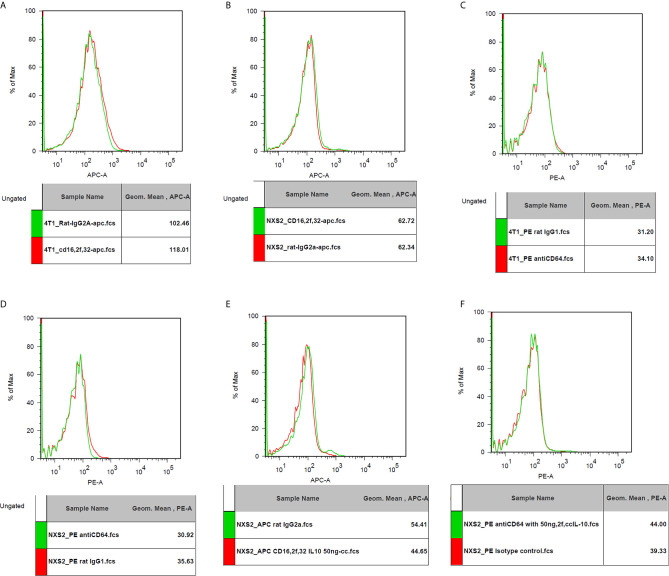
CD16/32 expression on 4T1 or NXS2 cells. **(A)**. CD16/32 Fc receptor expression on 4T1 cells compared to isotype control. There was no CD16/32 expression on 4T1 cells. **(B)** CD16/32 Fc receptor expression on NXS2 cells compared to isotype control. There was no CD16/32 expression on NXS2 cells. **(C)** CD64 Fc receptor expression on 4T1 cells compared to isotype control. There was no CD64 expression on 4T1 cells. **(D)** CD64 Fc receptor expression on NXS2 cells compared to isotype control. There was no CD64 expression on NXS2 cells. **(E)** CD16/32 Fc receptor expression on NXS2 cell treated by IL-10 50ng/cc for two days. There was no expression of CD16/32. **(F)** CD64 Fc receptor expression on NXS2 cell treated by IL-10 50ng/cc for two days. There was no expression of CD64.

**Figure 7:**
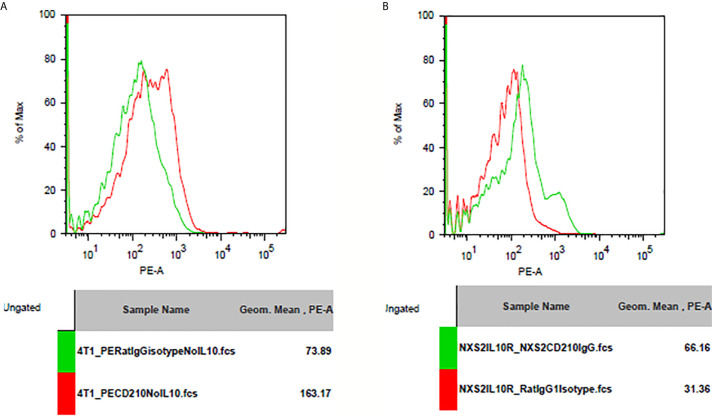
IL-10 receptor expression on 4T1 or NXS2 cells. **(A)** IL-10 receptor(CD210) expression on 4T1 cells. There was strong CD210 expression on 4T1 cells compared to isotype control. **(B)** IL-10 receptor(CD210) expression on NXS2 cells. There was strong CD210 expression on NXS2 cells compared to isotype control.

I used flowcytometry to estimate the expression of the Fc receptor (CD64, CD16, and CD32) and the IL-10 receptor on NXS2 and 4T1 cells and found that neither CD16/CD32 nor CD64 were expressed in NXS2 cells or 4T1 cells. Further, IL-10 treatment could not stimulate the expression of Fc receptors on NXS2 tumor cells. Surprisingly, IL-10 receptor expression was found both in 4T1 cells and NXS2 cells, and there were two subpopulations of 4T1 and NXS2 cells with different levels of IL-10 receptor expression. Thus, IL-10 might have a direct inhibitory effect on 4T1 and NXS2 cells *via* the IL-10 receptor (**Figures 6** and **7**).

### Induction of GD2 Expression in Immune Cells After IL-10 Treatment

**Figure 8:**
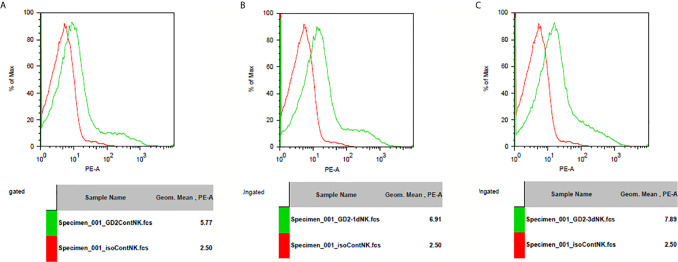
GD2 antigen expression on spleenocytes of mice. **(A)** GD2 antigen expression on spleenocytes from normal mice compared to isotype control. There was GD2 expression on spleenocytes of normal mice. **(B)** GD2 antigen expression on spleenocytes from mice injected with IL-10 for one day compared to isotype control. There was GD2 expression on spleenocytes of mice with one dose of IL-10. The magnitude of GD2 expression of one-dose IL-10 injected mice is greater than that of normal mice. **(C)** GD2 antigen expression on spleenocytes from mice injected with IL-10 for three days compared to isotype control. There was GD2 expression on spleenocytes of mice with three doses of IL-10. The magnitude of GD2 expression of three-dose IL-10 injected mice is greater than that of normal mice and greater than that of one-dose IL-10 injected mice.

GD2 antigen expression level was assessed after IL-10 treatment in splenocytes from Balb/c mice. I found that IL-10 induced the expression of GD2 antigen on immune cells. In addition, IL-10 treatment of mice for 1 or 3 days enhanced GD2 expression on their splenocytes compared to that on splenocytes from control mice (**Figure 8**).

## Discussion

In my study, I report that IL-10 has a significant effect on tumor shrinkage and survival in mice. Previous studies have reported that IL-10 mediates the immune response against the tumor and also inhibits metastasis in a mice model (7, 8). These findings motivate the use of IL-10 for cancer immunotherapy. Previous studies have provided contradictory evidence about the anti-tumor effects of IL-10. While some studies have suggested that IL-10 can suppress tumors, others have suggested that tumor-associated elevation of IL-10 can suppress the anti-tumor immune response of the host (9, 10). My findings support the first hypothesis, wherein we demonstrate that IL-10 exhibits strong anti-tumor effects. In clinical cancer patients, IL-10 is upregulated to initiate an anti-cancer immune response. However, this elevation of IL-10 may not be high enough to combat cancer, and thus, the authors could have drawn incorrect conclusions about the role played by IL-10 in the immune response against tumors.

Previous *in vitro* studies have reported that IL-15 activates NK and T cells and mediates anti-tumor immune responses (11, 12). During a viral infection, IL-15 is usually upregulated to induce proliferation of NK cells and memory T cells. In this study, we also found that IL-15 has a significant effect on tumor shrinkage and prolonging survival in mice. Although IL-15 is not as effective as IL-10, we demonstrated the potential of IL-15 in cancer immunotherapy. IL-15 belongs to the IL-2 cytokine family; however, in a previous study, IL-15 was shown to have a wider range of therapeutic effects with fewer side effect compared to those of IL-2, making IL-15 a good candidate for cancer treatment.

THαβ-mediated immune response involves IL-10-secreting CD4^+^ T cells (5). IL-10 plays a central role in mediating the THαβ-mediated immune response. Thus, I deduced that IL-10 is crucial in initiating the NK cell-mediated ADCC response, and this has also been suggested in a previous *in vitro* study. IL-10 and IL-15 are both vital immune mediators against viral infections, especially IL-10. IL-10 itself is the autocrine for IL-10-secreting CD4 T cells (Tr1 cells) in THαβ immunity (13). Although I didn’t isolate Tr1 cells in this investigation, several previous studies also point out the importance of IL-10-secreting Tr1 CD4 T cells for tumor immunity. In addition, IL-10 is a potent CD8 T cell activator, NK cell activator, and B cell activator with IgG1 class switch. THαβ do play a vital in tumor immunity.

IFN-γ is conventionally used for cancer therapy. However, IFN-γ treatment in cancer clinical trials has been shown to have limited success. Some studies have even reported that IFN-γ can worsen the prognosis of cancer patients. Surprisingly, I found that IFN-γ reduces survival and leads to an increase in tumor size in treated mice compared to that in control mice. IFN-γ is a major cytokine secreted by TH1 and activates monocytes/macrophages. In several previous animal studies, murine IFN-γ has been shown to increase metastasis of cancers in mice (14, 15). Since IFN-γ is a strong macrophage activator and can induce macrophage fusion, we suggest that IFN-γ not be used in cancer treatment strategies. Cancer metastasis is thought to be mediated by tumor-macrophage fusion (16), and after tumor-macrophage fusion, cancer cells are brought to macrophage reservoir pools in our body, such as in the brain (microglia), liver (Kupffer cells), spleen (spleen macrophages), bone (osteoclast), and lungs (alveolar macrophages). Tumor-macrophage fusion can also enhance angiogenesis and cancer invasiveness because a tumor can acquire Toll-like receptors from monocytic cells and proteases such as matrix metalloproteinases, which disrupts extracellular matrix (17–19). Thus, it is reasonable to suggest that IFN-γ is detrimental to host immune responses against cancer. The IL-10 receptor is only expressed on hematopoietic cells, including T cells, B cells, NK cells, mast cells, dendritic cells, and monocyte/macrophages, while Fc receptors are usually expressed in terminally differentiated macrophages (20). Thus, our results implied that cancer-macrophage fusion is actually a cancer-monocyte fusion, and fusion with terminally differentiated macrophages can inhibit cell proliferation (21). Besides, IL-10 is harmful to cancer cells and evolution process cannot let cancer to express IL-10 receptor to harm itself. It implies that cancer does have a monocyte origin to explain why cancer cells express IL-10 receptor. CD14 is a common marker of monocytic lineage, especially terminally differentiated macrophages. Although CD14 express level was not tested in this study, a previous report did point out the expression of CD14 antigen along with TLR2 and TLR4 are present on 4T1 cells (22). CD14 and CD16 are important monocyte markers in human. However, CCR2 and CX3CR1 are more important monocyte markers in mice. Previous study found that CCR2 is over-expressed on 4T1 cells, and CX3CR1 is over-expressed on NXS2 cells (23, 24). These data imply that cancers have a monocyte origin. The cancer cell lines from our study thus must have originated from fused monocytic cells (25).

The first tumor model investigated in this study was a murine model of breast cancer. However, unlike human breast cancer, murine breast cancers do not have the Globo H antigen, so I was unable to use a VK9 anti-Globo H antibody to treat the 4T1 mice breast cancer model (data not shown) (26). My second tumor model was a murine model of neuroblastoma. My original hypothesis is that IL-10 plus anti-GD2 antibody may activate the NK cell-mediated ADCC to kill cancer cells (27). Surprisingly, we found that IL-10 plus anti-GD2 antibody treatment was not significantly better than IL-10 alone. IL-10 treatment groups had the longest survival and the smallest tumor volume. Previous studies have suggested that tumor cells can express the Fc IgG receptor to bind and interfere with the antibody-mediated elimination of the tumor (28, 29). However, I found that the CD64/CD32/CD16 Fc receptor was not expressed on 4T1 and NXS2 cells. A previous study reported that GD2 expression is found on lymphocytes (30), and therefore, we estimated GD2 expression on the splenocytes/immune cells of mice and found strong GD2 expression on these immune cells. Thus, an anti-GD2 antibody could attack these immune cells and inhibit the anti-tumor immune response, and this deduction helps to explain our results. In addition, I found that both 4T1 and NXS2 cells have a strong IL-10 receptor expression, and IL-10 is directly toxic to both 4T1 and NXS2 cells *in vitro*. Furthermore, IL-10 can kill monocytes/macrophages (31), suggesting that IL-10 can directly kill tumor cells as well as tumor-associated macrophages. This study provides the first evidence that IL-10 can directly kill cancer cells, thus demonstrating that IL-10 is a highly effective anti-tumor agent. A previous study found that the anti-tumor effect of IL-10 is IFN-γ dependent (32). And, that was a rationale for conducting an IL-10 cancer treatment phase 1 trial (33). In this study, I found that direct tumor and tumor associated macrophage inhibition should be the actual reason of the efficacy of IL-10. We know the microenvironment of cancers are critical to cancer proliferation and progression. Tumor associated macrophages, the prominent population in the tumor mass, are crucial to cancer progression. Although I did not dissect the tumor microenvironment in this study, the suppressive effect of IL-10 on tumor associated macrophages does help to stop cancer progression. Another pancreatic cancer clinical trial suggested that IL-10 combined with FOLFOX (Leucovorin, 5-FU, and Eloxatin) has no survival benefits (30). However, that study did not exclude the possibility of the toxicity of chemotherapeutic agents. IL-10 treatment for solid tumors is still very promising.

In summary, THαβ-mediated immune response is an effective anti-tumor response. Both IL-10 and IL-15 have significant effects on tumor volume shrinkage as well as on prolonging mice survival. However, IFN-γ appears to have negative effects on tumor immunity; it enhances tumor growth and reduces mice survival rate. IL-10 has direct anti-tumor and anti-macrophage toxicity *in vitro*, reinforcing its effectiveness in mediating anti-tumor immune responses. My findings strongly warrant the formulation of a clinical trial to test the anti-tumor efficacy of IL-10 in oncology patients in the near future.

## Data Availability Statement

The raw data supporting the conclusions of this article will be made available by the author, without undue reservation.

## Ethics Statement

The animal study was reviewed and approved by Institutional Animal Care & Use Committee, Academia Sinica.

## Author Contributions

The author confirms being the sole contributor of this work and has approved it for publication.

## Funding

This study was funded by Academia Sinica, Taiwan (High Peak Project) and Taipei Tzu Chi Hospital (TCRD-TPE-109-65).

## Conflict of Interest

The author declares that the research was conducted in the absence of any commercial or financial relationships that could be construed as a potential conflict of interest.
